# Examining the Relationship Between Cultural Identity, Cultural Stress, and Mental Health Outcomes in Recently Immigrated Venezuelan Families

**DOI:** 10.3390/bs15081110

**Published:** 2025-08-16

**Authors:** Carolina Scaramutti-Gladfelter, Tae Kyoung Lee, Seo Woo Lee, Elena Bochkina, Alejandra García Isaza, Pablo Montero-Zamora, Mariano J. Kanamori Nishimura, Eric C. Brown, Seth J. Schwartz

**Affiliations:** 1 Department of Psychiatry and Behavioral Sciences, School of Medicine, University of Miami Miller, Miami, FL 33136, USA; 2 Social Innovation Convergence Program, Sungkyunkwan University, Seoul 03063, Republic of Korea; ltk501@skku.edu; 3 College of Education, The University of Texas at Austin, Austin, TX 78712, USA; cldhkdhk@utexas.edu (S.W.L.); alejandra.garciaisaza@utmail.utexas.edu (A.G.I.); pmontero@austin.utexas.edu (P.M.-Z.); seth.schwartz@austin.utexas.edu (S.J.S.); 4 Department of Psychology, Moscow City University, 119991 Moscow, Russia; bochkina.elena@gmail.com (E.B.); mkanamori@med.miami.edu (M.J.K.N.); ricbrown@miami.edu (E.C.B.); 5 Department of Public Health Sciences, School of Medicine, University of Miami Miller, Miami, FL 33136, USA

**Keywords:** crisis migrants, actor–partner interdependence model, cultural stress

## Abstract

This study explores how ethnic identity, national identity, and cultural stress interact to impact mental health among Latino youth and their parents, using the Actor–Partner Interdependence Mediation Model (APIMeM). By analyzing survey data from Latino parent–youth dyads, we assessed both individual (actor) and cross-dyadic (partner) effects of identity and cultural stress on mental health outcomes. The APIMeM framework allowed us to examine whether cultural stress mediates the relationship between identity factors and mental health. Results revealed significant actor effects, with higher levels of cultural stress associated with increased mental health distress in both parents (β = 0.65, *p* < 0.001) and youth (β = 0.32, *p* < 0.001). Ethnic identity did not demonstrate significant actor or partner effects on either cultural stress or mental health. In contrast, stronger national identity was inversely related to youth mental health distress (β = −0.11, *p* = 0.01) and had a significant protective partner effect on parental mental health (β = −0.16, *p* = 0.02). However, cultural stress did not mediate the relationship between ethnic identity and mental health. These findings underscore the importance of national identity and interdependent family dynamics in shaping mental health outcomes within Latino families experiencing cultural stress.

## 1. Background

Over the past several decades, the world has experienced a notable increase in migration, spurred by myriad socio-political, economic, and environmental forces. This surge in movement across borders is rooted in increases in climate-related disasters, invasions, civil wars, governmental collapses, and other types of natural and human disasters. One such migration wave involves Venezuelans migrating to the United States (as well as to other Latin American countries) following the collapse of their government and of much of their society (Salas-Wright et al., 2022). The Venezuelan migration is unique because it does not involve a natural disaster or armed conflict—rather, individuals are fleeing government repression, economic stagflation, and violence that has exponentially increased the difficulties involved in obtaining food, medication, and other necessities.

McAdam (2014) coined the term *crisis migration* to refer to large numbers of individuals and families leaving the epicenter of a disaster hastily and settling in various destination countries and regions. Crisis migrants may be especially likely to encounter cultural stressors, such as discrimination and negative context of reception (i.e., exclusion from opportunities and social networks in the destination country or region) upon arrival (e.g., Schwartz et al., 2025). These individuals can draw upon key assets such as cultural identity—identification with their countries of origin and with the destination country or region—to help offset the effects of cultural stressors on their mental health.

It is also essential to note that many crisis migrants—including Venezuelans—arrive in their destination countries as family units (one or both parents with one or more children). As a result, it is essential to examine family processes within Venezuelan crisis migrant samples. Family systems theory (Bowen, 1978) holds that one family member’s experiences, perceptions, or beliefs are likely to be associated with mental health (and other) outcomes among other family members. In other words, families function as dynamic and interdependent systems, where each family member’s thoughts, feelings, and behaviors can affect those of other family members. A key example involves parents and children, where parents’ cultural stressors can predict youth outcomes (Lorenzo-Blanco et al., 2016) and vice versa. For another example, parents’ ethnic and United States identity might predict cultural stress and mental health outcomes among youth—and vice versa.

Actor–partner interdependence modeling, as well as other types of dyadic modeling, represents a potentially useful way of modeling this within-family interplay (Wolff et al., 2020). Dyadic modeling, where parents and youth are considered as nested within larger family units, maps analytically onto family systems theory. Indeed, youth-reported and parent-reported variables are allowed to predict one another, as happens within families—and parent and youth outcome variables (such as depressive symptoms and anxiety) are specified as correlated with one another. It is also possible to specify mediating mechanisms through which parent- and youth-reported predictor variables may influence one another—and the result is Actor–Partner Interdependence Mediation Modeling (APIMeM; Ledermann et al., 2011).

Crisis migrants face many of the same challenges that other migrant groups face, but they may have fewer physical and financial resources at their disposal to address these challenges due to their need to move without much planning. Migrants moving with their families have increased responsibility for children and limited capacity to deal with problems that arise during and after migration. Consequently, they may experience guilt and somatic symptoms of shame (Bhugra, 2003), which may depend on migrants’ culture of origin—such that depressive symptoms may go undetected. However, as Cobb et al. (2019) and Salas-Wright et al. (2021) have noted and found, many crisis migrants are flourishing and thriving in their new environments, especially in countries with developed economies where job opportunities are available. In the next subsection, we review the concept of cultural identity and how it can serve as a key resource for Venezuelans and other crisis migrant groups.

### 1.1. Cultural Identity

Broadly speaking, cultural identity refers to an individual’s connection to a group with shared beliefs, customs, and practices. Cultural identity is often, but not always, circumscribed to racial, ethnic, or geographical categories (Unger, 2011). Cultural identity is a multidimensional construct encompassing both ethnic and national identity. Ethnic identity refers to an individual’s sense of belonging to a particular cultural group, shaped by shared ancestry, language, traditions, and values. National identity, on the other hand, reflects one’s attachment to the country or region where one lives, and is often influenced by citizenship, political structures, and collective national experiences (Schildkraut, 2014). Nonetheless, as individuals and families traverse geographical borders, questions of ethnic and national identity become paramount as components of cultural identity, with considerations of heritage, language, and traditions influencing one’s sense of self within the larger societal framework. In essence, cultural identity refers to the question of “*who am I as a cultural being?*” For migrants and other bicultural individuals, this question inherently involves psychological attachments to the cultural heritage one brings from one’s country of origin, along with the destination country’s customs and way of living. Migration inherently triggers a renegotiation of cultural identity, presenting Venezuelan migrant families with many considerations. They grapple with preserving their Venezuelan heritage while settling into life in the United States. Simultaneously, exploration and expansion of national identity unfolds as individuals and families consider what living in the United States means to them—as well as the extent to which they consider themselves to be members of society in the United States.

Because many migrant individuals and families become bicultural following migration, ethnic and national identity often operate simultaneously. Put differently, rather than representing opposing ends of a single dimension, ethnic and national identity are often positively interrelated and may predict many of the same outcomes. As a result, ethnic and national identity must be assessed separately and used together as predictors of mental health and cultural stress outcomes.

Such knowledge helps migrants understand social norms, language, and expectations in their new environment while maintaining a connection to their heritage—thus reducing cultural dissonance and enhancing their ability to adapt (Portes & Rumbaut, 2014). For example, being able to speak both English and Spanish and understanding both Venezuelan and U.S. cultural systems likely helps migrants to navigate both sets of cultural spaces. Additionally, by retaining their heritage–cultural identity, Venezuelan migrants can build social support networks, preserve traditions, and develop bicultural competence, which is associated with better psychological and socio-economic outcomes during the acculturation process (Nguyen & Benet-Martínez, 2013).

### 1.2. Cultural Stress Theory

Cultural stress theory provides important information for understanding the psychological toll experienced by Venezuelan families following arrival in the United States. For Venezuelan families, navigating the unfamiliar cultural landscape of the United States can introduce a myriad of stressors. Among these stressors are discrimination and negative context of reception. Here we define *discrimination* as name-calling, slurs, mocking immigrants’ accents, threats, and intimidating them (Corral & Landrine, 2008). Discrimination, whether overt or covert, in addition to facing barriers to or diminished opportunities, can create chronic stress and feelings of unworthiness or rejection. *Negative context of reception* is defined as being denied opportunities and feeling unwelcome in the destination country (Portes & Rumbaut, 2014). Aspects of negative context of reception—such as restrictive immigration policies, limited access to healthcare, or lack of community support—can heighten uncertainty, fear, and hopelessness, further impacting psychological well-being. Experiencing discrimination and a negative context of reception can be disturbing and unsettling—especially for families who have fled crisis situations and who thought they would be “safe” after arriving in the United States. In addition, discrimination and negative context of reception often co-occur with one another to form a latent cultural stress construct (Schwartz et al., 2015).

Cultural stressors are also commonly associated with compromised mental health (see Salas-Wright & Schwartz, 2019, for a review). In the present article, we define compromised mental health in terms of depressive and anxiety symptomology. It is critical to consider depressive and anxiety symptoms as they are associated with billions of dollars per year in treatment, missed work, and maladaptive coping strategies such as alcohol and drug use (Greenberg et al., 2021; Substance Abuse and Mental Health Services Administration, 2024). Previous studies indicated significant positive correlations between symptoms of depression and anxiety (e.g., Cohen et al., 2018).

Although cultural stressors and their associations with mental health have been examined among various migrant groups, Venezuelans are an important population to which these findings need to be extended. Largely due to the collapse of their country under the Maduro regime, Venezuelans are one of the fastest-growing Latin American migrant groups in the United States (Pew Research Center, 2023). Venezuelans also often experience considerable trauma prior to and during their migration journey (Duque et al., in press)—including street violence, food insecurity, and perilous journeys through several countries to reach the U.S. border. The majority of research with Latin American populations in the United States has been conducted with Mexicans, Puerto Ricans, and Cubans—such that South American groups have been considerably understudied. Because Venezuelans are a new and rapidly increasing population, examining cultural stressors and mental health among this population is an essential research direction.

Cultural stress may be especially important to study within families (see Lorenzo-Blanco et al., 2016). Cultural stressors do not necessarily affect all family members equally, and they may contribute to intergenerational conflict within migrant families. Parents and children often acculturate at different rates, potentially leading to disagreements about values, behaviors, and expectations. These cultural conflicts within families can contribute to strained relationships, emotional distress, and difficulties in maintaining a cohesive family unit. Further, parents’ cultural stressors can predict impaired youth mental health because parents who are culturally stressed may have a difficult time staying positively involved with their children. Parents’ and youth’s cultural stressors may also predict *one another’s* mental health outcomes (Ertanir et al., 2024)—further stressing the importance of studying cultural stress and mental health within family systems.

Despite these challenges, protective factors such as cultural identity, social support, and access to culturally responsive mental health services can buffer the negative effects of cultural stress. Understanding the nuanced ways in which cultural stress impacts Venezuelan migrants can inform targeted interventions aimed at promoting resilience, mental health, and successful adaptation in their new environment.

### 1.3. The Present Study

The present study was designed to examine the mediating role of cultural stress in the predictive effects of ethnic and national identity on mental health outcomes among Venezuelan immigrant parent–youth dyads. We examined the entire predictive sequence from a dyadic, family-systems perspective, where both youth and parent cultural identity were allowed to predict both youth and parent cultural stress and mental health. In terms of hypotheses, we expected that across reporters a mediation effect would emerge whereby cultural identifications would negatively and indirectly predict symptoms of anxiety and depression through discrimination and negative context of reception. We expected that this mediation effect would emerge both between and across reporters.

The present article consists of nine authors. Two authors are Hispanic females, two are Hispanic males, one is an Asian female, one is an Asian male, one is a White female, one is a White male, and one is a male of mixed ethnic heritage. The first author, a Hispanic female, conceptualized the study, collected the data, conducted the analyses, and led the writing of the manuscript. Other authors collaborated on analyses and writing.

## 2. Methods

### 2.1. Participants

The present cross-sectional study was conducted in December 2023, with 103 Venezuelan parent–child dyads living in the United States. To be eligible, families had to indicate that they (1) had arrived from Venezuela to the United States in the last 3 years; (2) had a youth between the ages of 12 and 18 willing to participate; (3) currently resided in the United States.

Our study sample consisted of 103 Venezuelan parent–youth dyads. All participants were residing in the United States at the time of data collection. Data were collected at one time point as an anonymous survey with only a dyad number assigned to each parent–child pair. Data were collected from both the parent and the youth. The majority of families (*n* = 92) had resided in the United States for 24 months or less. Parents reported working, on average, between 41 and 60 h per week. Our sample consisted mainly of girls and women (*n* = 70 for adults, *n* = 65 for youth). The majority of participants had resided in the United States for 24 months or less.

### 2.2. Procedures

Families were recruited in Miami (the United States city with the largest concentration of Venezuelan immigrants). Participants were recruited through a community partner, Raices Venezolanas, in Miami. A flyer was provided to Raíces Venezolanas and was posted on their social media page. Participants were provided with an incentive for completing the online survey. Surveys were anonymous, and a dyad ID was provided to each parent–child pair.

Parents interested in participating emailed the recruiter, who verified whether they met the inclusion criteria. They also verified that the youth was willing to participate. Only one parent from each household was invited to participate for each family that met inclusion criteria. If a family had multiple youths within the 12 to 18 age range, one child was selected randomly to participate. A $15 gift card was provided as an incentive for each participating adult and youth.

### 2.3. Measures

*Sociodemographics.* Sociodemographic variables included biological sex (1= male, 0= female), age in years, years residing in the United States, and partnered status (1= yes, 0 = no).

*Ethnic Identity.* We assessed parent and youth ethnic identity using the Multigroup Ethnic Identity Measure (Roberts et al., 1999). This measure looks at one’s exploration of and commitment to a sense of ethnic identity. A 5-point response scale was used to index how much each person agreed or disagreed with each statement, with response choices ranging from 1 (*Strongly disagree*) to 5 (*Strongly agree*). Items were summed to create a total score. Cronbach’s alphas in the present sample for parents and children at baseline were 0.87 and 0.94, respectively.

*National Identity.* The American Identity Measure (Schwartz et al., 2012) was developed by adapting Phinney’s multi-ethnic identity measure. Specifically, “the United States” was inserted in place of “my ethnic group”. A 5-point Likert scale, ranging from 1 (*Strongly Disagree*) to 5 (*Strongly Agree*), was used to record responses. Cronbach’s alphas in the present sample for parents and children at baseline were 0.90 and 0.90, respectively.

*Cultural Stress.* We assessed two kinds of perceived cultural stressors: (a) discrimination and (b) negative context of reception. We assessed perceived discrimination using a seven-item scale developed by Phinney et al. (1998). Items focus on the frequency of discrimination incidents that migrants may experience in a new environment (e.g., being called offensive names because they are Venezuelan/Hispanic). A 5-point response scale was used to measure how often each event occurred from 1 (*Never*) to 5 (*Almost every day*). Items were summed to create a total score. Cronbach’s alphas in the present sample for parents and children at baseline were 0.70 and 0.93, respectively.

We also used the Negative Context of Reception Scale (Schwartz et al., 2014) to assess perceived negative context of reception. This 6-item scale assesses how Venezuelans perceive differences in opportunities and reception in the United States context compared to other migrant groups (e.g., “People in this country often criticize people from Venezuela”). Items were rated using a 5-point scale ranging from 1 (*Strongly disagree*) to 5 (*Strongly agree*). Cronbach’s alphas in the present sample for parents and children at baseline were 0.85 and 0.88, respectively.

*Mental health.* We assessed two kinds of psychological distress: (a) anxiety and (b) depressive symptoms. We assessed parent and youth anxiety using the Generalized Anxiety Disorder Scale (GAD; Spitzer et al., 2006). This 7-item scale was used to assess symptoms of anxiety during the two weeks prior to assessment. The GAD assesses symptoms such as excessive worrying, tension, irritability, and difficulty sleeping. Items were rated on a scale ranging from 0 (*not at all*) to 3 (*nearly every day*). Items were summed to a total score. Cronbach’s alphas in the present sample for parents and children at baseline were 0.85 and 0.92, respectively.

We used the 10-item version of the Center for Epidemiological Studies Depression Scale (CESD-B; Grzywacz et al., 2010) to assess symptoms of depression among parents and youths. The 10-item CESD-B taps into symptoms such as tiredness, loneliness, and sadness. The scale includes 8 negative items (e.g., “*I felt that everything I did was an effort*”) and 2 positive items (e.g., “*I felt hopeful about the future*”). Items were rated using a 5-point response scale ranging from 1 (*rarely or none of the time*) to 4 (*most or all of the time*). Items were summed to create a total score, with higher values representing greater depressive symptomatology. Cronbach’s alphas in the present sample for parents and children at baseline were 0.77 and 0.88, respectively.

## 3. Analysis

In the present study, we evaluated a mediational model where ethnic and national identity would predict mental health outcomes (symptoms of depression and anxiety) indirectly through cultural stress (discrimination and negative context of reception).

**Figure 1:**
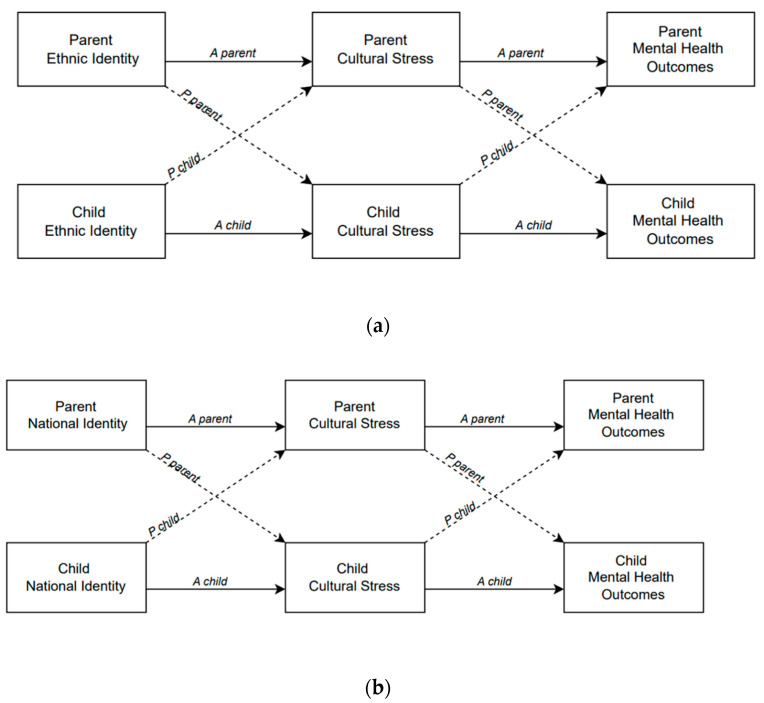
(**a**) APIMeM model depicting actor (A) and partner (P) effects among ethnic identity, cultural stress, and mental health in parent–child dyads. (**b**) APIM model depicting actor (A) and partner (P) effects among national identity, cultural stress, and mental health in parent–child dyads.

Our model also adopts a dyadic, family-systems perspective by examining the mediational sequence (a) within parents, (b) within youth, (c) with parents’ cultural identification predicting youth cultural stress and mental health, and (d) with youth cultural identification predicting parents’ cultural stress and mental health. We were able to determine the ways in which parent and youth cultural processes predicted their own, and one another’s cultural stress and mental health (see Figure 1a,b).

### 3.1. Descriptive Statistics and Bivariate Correlations

Demographic information was computed using IBM SPSS Statistics (Version 27). Model fit, as proposed by Hancock et al. (2000), was assessed using the Chi Square, Comparative Fit Index (CFI), Tucker–Lewis Index (TLI), Root Mean Square Error of Approximation (RMSEA), and Standard Root Mean Square Residual (SRMR) indices. Acceptable model fit is represented as CFI ≥ 0.95, TLI ≥ 0.95, RMSEA ≤ 0.08, and SRMR ≤ 0.06 (Kline, 2023). The Chi-square index is reported but is not used in interpretation because it tests the null hypothesis of perfect model fit—which is tenable only in the simplest models.

### 3.2. Hypothesized Model Estimation

The following two distinct models were tested: one featuring ethnic identity as the independent variable, cultural stress as the mediating variable, and mental health as the outcome; and the second with national identity as the independent variable, cultural stress as the mediating variable, and mental health as the outcome.

To explore the role of cultural stress as a mediating factor, we utilized discrimination and the negative context of reception to formulate the latent cultural stress variable. The correlation was strong enough between these two indicators to support a latent variable (*r* = 0.60, *p* < 0.001). Indicators for the latent mental health variable included symptoms of depression and anxiety. We conducted tests of measurement invariance to assess whether factor loadings and item intercepts were equivalent between parents and adolescents.

To validate the latent constructs, we initially utilized Confirmatory Factor Analysis (CFA) within the framework of the Actor–Partner Interdependence Mediation Model (APIM-eM). CFA assesses the alignment of observed variables (indicators) with the hypothesized latent constructs. Using the same indicators for latent variables across parents and children, we conducted three tests for measurement invariance to determine whether these indicators patterned consistently onto their respective latent variables across both reporters.

Traditional invariance testing involves assessing three levels—configural, metric, and scalar (Putnick & Bornstein, 2016). Configural invariance entails estimating a model without cross-reporter constraints on factor loadings or item intercepts. If the configural invariance model fits the data well, we would proceed to estimate a metric invariance model where each factor loading is constrained to be equal across reporters. The assessment of metric invariance involves comparing the fit of the constrained (metric) model to the unconstrained (configural) model. If the model fit does not significantly decrease when these constraints are applied, we can retain the assumption of metric invariance. Finally, scalar invariance (equal item intercepts across reporters) is examined by comparing the fit of the metric invariance models against a scalar invariance model, where both loadings and intercepts are constrained to be equal across reporters. Again, if there is equivalent fit between the metric and scalar invariance models, we can retain the assumption of scalar invariance. Each model test is conducted by comparing CFI and RMSEA values between models, with differences of |0.01| or less in both CFI and RMSEA indicating equivalent fit. The Chi-square difference test is reported but is not used in interpretation due to its tendency to be overpowered and suggest significant differences in model fit even when differences in the fit indices themselves are minimal.

### 3.3. Confirmatory Factor Analysis

We observed robust and positive factor loadings for depression and anxiety on the mental health latent variable for both parents and adolescents (average factor loading = 0.75). This finding underscores their validity as effective indicators of mental health outcomes. Next, we performed the configural invariance test to validate the presence of uniform latent factors among parents and youth. The configural model, where no constraints were imposed on model parameters, displayed a commendable fit to the data, as indicated by the following favorable fit indices: RMSEA = 0.067, CFI = 1.00, TLI = 1.00, and SRMR = 0.03. Factor loadings for discrimination and the negative context of reception on the cultural stress latent variable were consistently positive across reporters, as outlined in Table 1.

The metric model displayed acceptable fit to the data, as indicated by the following fit indices: RMSEA = 0.069, CFI = 1.00, TLI = 1.00, and SRMR = 0.03. These values are not different enough (DCFI ≥ 0.010 and/or DRMSEA ≥ 0.010) from the fit of the configural model to reject the null hypothesis of metric invariance. The model fit for metric invariance was deemed acceptable, thus providing justification to proceed with the evaluation of full scalar invariance.

In the assessment of scalar invariance, item intercepts were constrained to be equal between parents and youth reports. Consequently, it yielded a well-fitting model: RMSEA = 0.073, CFI = 1.00, TLI = 1.00, and SRMR = 0.04. Again, these values are not different enough (DCFI ≥ 0.010 and/or DRMSEA ≥ 0.010) from the fit of the metric model to reject the null hypothesis of scalar invariance. Therefore, this configuration was retained for subsequent analysis. These results support the idea that the fundamental factor structure remains stable across both groups, indicating the presence of the same latent construct in both parents and youths.

### 3.4. Mediation Analysis

To evaluate our study hypotheses, Actor–Partner Interdependence Mediation Modeling (APIM-eM; Kenny et al., 2006) was employed. This technique allows the simultaneous testing of predictor effects within a dyad on individual family members’ outcomes (actor effect) and on their partner’s outcomes (partner effect). Structural equation modeling with maximum likelihood estimation for distinguishable dyads (Stas et al., 2018) was used within the APIM-eM analyses. Skewness and kurtosis within the acceptable range (−2 to +2) were verified for each variable so that we could treat all study variables as continuous.

In a dyadic context, actor mediation effects refer to *intra-individual direct* and *indirect effects* (e.g., direct effect: parent’s national identity → parent’s mental health; indirect effect: parent’s national identity → parent’s cultural stress → parent’s mental health; see Figure 1a,b). Partner mediation effects refer to *inter-individual direct and indirect effects* (e.g., direct effect: parent’s ethnic identity → youth’s mental health; indirect effect: parent’s ethnic identity → youth’s cultural stress → youth’s mental health).

Due to our small sample size, we opted to use factor scores for each latent variable. This approach allowed us to create a manifest variable capturing each underlying construct, thereby reducing the number of parameters requiring estimation (Devlieger et al., 2016). These factor scores, also termed factor-based composite scores, are obtained from a factor analysis of observed variables. The process of generating factor scores includes two main steps: firstly, estimating the measurement model for each latent factor individually within a structural regression model, and extracting factor scores for each variable; secondly, utilizing these factor scores as input data in a subsequent ordinary least squares (OLS) regression or path analysis model. In the context of our study, the observed constructs for the cultural stress latent variable include discrimination and negative context of reception. Likewise, for the mental health latent variable our observed constructs are depression and anxiety. We derived factor scores for each latent variable using the factor loadings obtained from this analysis. Subsequently, these factor scores served as indicators for our latent constructs within the Actor–Partner Interdependence Mediation Model (APIM-eM).

Next, we conducted a mediation analysis (MacKinnon, 2008) to explore the indirect predictive impact of ethnic identity (or national identity) on mental health outcomes through cultural stress. Employing a dyadic structural equation model, we examined the mediating role of cultural stress in the connection between ethnic (or national) identity and mental health outcomes. Given our relatively small sample size, we employed bootstrapping with 10,000 iterations to assess the extent to which cultural stress mediates the predictive effect of identity on mental health outcomes. Bootstrapping, a resampling technique involving repeated sampling with replacement from the observed data, was used to increase statistical power. A significant indirect effect was inferred if the corresponding 95% bias-corrected bootstrap confidence interval (CI) did not include zero.

## 4. Results

### 4.1. Actor–Partner Interdependence Mediation Model (APIMeM) Analysis

#### 4.1.1. Direct Effects (Actor)—Ethnic Identity

We assessed the direct effects within the APIM-eM. For actor effects of ethnic identity on cultural stress, results indicated no significant actor effects for either parents or youths (β = 0.13, *p* = 0.24 for parents; β = 0.03, *p* = 0.85 for youths). Additionally, we assessed the direct actor effects of ethnic identity on mental health. Results indicated no significant actor effect for either youths or parents (β = 0.003, *p* = 0.94 for youths; β = 0.12, *p* = 0.19 for parents).

We assessed the direct actor effects of cultural stress on mental health. Results indicated a significant positive actor effect for parents (β *=* 0.65, *p* < 0.001), indicating that higher levels of cultural stress were associated with higher levels of mental health distress. For youths, results indicated a significant positive actor effect (β = 0.32, *p* < 0.001), indicating that higher levels of cultural stress predicted higher levels of mental health distress.

#### 4.1.2. Direct Effects (Partner)—Ethnic Identity

We assessed the direct effects of the individual’s ethnic identity on their partner’s cultural stress. There were no significant results for either parents or youths (Parent → Youth β = 0.04, *p* = 0.72; Youth → Parent β = −0.06, *p* = 0.63).

We then assessed the direct partner effects of each individual’s ethnic identity on their partner’s mental health. Results indicated no significant partner effects for parents or youths (Parent → Youth: β = 0.09, *p* = 0.10; Youth → Parent: β = 0.01, *p* = 0.86).

Lastly, we assessed the direct effects of each individual’s cultural stress on their partner’s mental health distress. Results indicated a significant positive predictive effect of parent cultural stress on youth mental health (β = 0.63, *p* < 0.001). There were also significant findings for the effect of youth’s cultural stress on parents’ mental health (β = 0.32, *p* < 0.001).

#### 4.1.3. Direct Effects (Actor)—National Identity

We assessed the direct effects within the APIM-eM. For actor effects, we examined the effects of national identity on cultural stress. Results indicated no significant actor effects for parents or for youths (β = 0.11, *p* = 0.20 for parents; β = −0.15, *p* = 0.14 for youths). Additionally, we assessed the direct actor effects of national identity on mental health. Results indicated no significant actor effect for parents (β = 0.03, *p* = 0.72). There was an inverse significant direct actor effect for youths (β = −0.11, *p* = 0.01), where higher national identity was associated with lower levels of mental health distress.

#### 4.1.4. Direct Effects (Partner)—National Identity

We assessed the direct effects of the individual’s national identity on their partner’s cultural stress. There were no significant results for either parents or youths (Parent → Youth β = 0.09, *p =* 0.26; Youth → Parent β = −0.06, *p* = 0.54). We then assessed the direct partner effects of each individual’s national identity on their partner’s mental health. Results indicated no significant partner effects for parents (Parent → Youth: β = 0.01, *p* = 0.82). There were significant inverse partner effects for youths (Youth → Parent: β = −0.16, *p* = 0.02).

### 4.2. Mediation Analysis

To investigate mediation, we tested whether cultural stress mediated the relationship between ethnic identity and mental health. Results indicated no significant mediated effects (see Table 2).

## 5. Discussion

The demographic characteristics of our study sample, comprising 103 Venezuelan parent–youth dyads, offer a valuable snapshot of the experiences of recent Venezuelan immigrants in the United States. The temporal aspect of their migration experiences is particularly noteworthy, as most participants had lived in the United States for 24 months or less. This temporal emphasis suggests the acute nature of their transition and settlement, offering a timely exploration into the initial phases of their acculturation and adaptation processes within the United States context.

We found no significant actor effects for national identity on cultural stress for either parents or youths. These nonsignificant actor effects imply that, at least within our sample, individual levels of national identity may not directly predict the experience of cultural stress for either parents or youths. However, we did find that higher national identity was associated with lower levels of mental health distress among the youth participants. This finding suggests that, for the youth participants, a stronger connection to the destination country (the United States) may serve as a protective factor against mental health challenges. This finding suggests that integration into the destination country’s culture is protective for the youth’s mental health. This finding could be rooted in a variety of mechanisms, such as fostering a sense of belonging, providing a stable anchor amidst the acculturation process, or offering a source of resilience through cultural pride and connection to heritage. Another explanation may be that feeling connected to the destination country helps immigrants develop a stronger sense of belonging within the destination society, fostering a more coherent personal identity (Schwartz et al., 2018). In turn, a more coherent sense of personal identity may help to reduce mental health distress.

Interestingly, significant actor effects were identified in the case of cultural stress influencing mental health distress. Both parents and youths displayed positive actor effects, implying that higher levels of cultural stress were predictive of higher levels of mental health distress for both groups. This observation suggests the pivotal role that cultural stress may play in shaping the psychological well-being of Venezuelan immigrant parent–youth dyads in the United States. For parents and youths alike, experiencing heightened cultural stress appears to contribute to a subsequent increase in mental health distress. This similarity raises important questions about the specific stressors associated with the acculturation process and how these challenges manifest in mental health concerns for both generations. The findings suggest that interventions aimed at mitigating cultural stress, either through promoting coping skills or through creating systemic change, may yield potential benefits for mental health outcomes among both parents and youths. Addressing stress related to acculturation, discrimination, or a negative context of reception could be a crucial addition to evidence-based mental health interventions to foster improved mental health outcomes for this immigrant population.

However, much cultural stress among Venezuelan immigrant families may not be directly addressable. Although the present data were collected in December 2023, it is worth noting that cultural stress for Venezuelans has increased since the second Trump administration was inaugurated in January 2025. The administration has deported many Venezuelans and has moved to end temporary protected status for hundreds of thousands of Venezuelans living in the United States (Quinn, 2025). Indeed, the administration has conflated asylum-seeking Venezuelan families with violent Tren de Aragua gang members and seeks to deport all Venezuelans who are not U.S. citizens or green-card holders—which by definition the participants in our study are likely not.

It is also important to note that cultural stress for Venezuelan families has been even higher in other South American countries, such as Colombia, than in the United States (Salas-Wright et al., 2024). As a result, with the Trump administration moving to expel Venezuelans from the United States, there may not be many other “friendly” destinations where they can settle. The present results suggest that mental health for many Venezuelan families will, therefore, likely be negatively impacted by these increases in cultural stress.

Moving on to partner effects, no significant direct effects were observed for ethnic or national identity on partner cultural stress or mental health, except for an inverse effect of youth’s national identity on parental mental health distress. This absence of direct partner effects suggests that, at least in this context, an individual’s identity—be it ethnic or national—may not exert a direct influence on the stress or mental health experiences of their partner. However, the finding regarding significant partner effects of youth national identity suggests that higher levels of national identity among the youths are associated with lower levels of mental health distress experienced by their parents. This particular partner effect introduces a unique narrative, emphasizing the potential cross-generational impact of a strong connection to national identity. Adolescent national identity plays a crucial role in shaping both their own psychological well-being and that of their parents, highlighting its significance beyond individual self-concept. As adolescents navigate cultural and societal expectations, a strong sense of national belonging can foster stability and resilience, serving as a protective factor against stressors such as discrimination and sociopolitical marginalization. This identity not only influences their own mental health (actor effect) by shaping their perceptions of acceptance and integration but also impacts their parents’ well-being (partner effect) by reinforcing family cohesion and shared cultural narratives. When adolescents hold a strong national identity parents may experience reduced distress as they perceive greater security and social acceptance for their children, thereby strengthening the overall family unit.

Further, mediation analyses exploring the role of cultural stress as a mediator between ethnic identity and mental health did not yield any significant findings. The utilization of a single time point for data collection is a crucial factor that may influence the observed lack of significant indirect effects. Given the dynamic nature of immigrant experiences, utilizing a one-time assessment may capture only a snapshot of the complex and evolving processes associated with acculturation, identity formation, and mental health outcomes. Ethnic identity, cultural stress, and mental health are constructs that can undergo changes and adaptations over time, and a singular measurement may not fully capture the nuanced interplay among these variables. It is recommended for future studies to examine actor and partner associations in immigrant families over an extended period. Longitudinal studies that track participants across multiple time points would offer a more comprehensive understanding of the temporal dynamics involved. Such an approach could capture fluctuations in ethnic identity, variations in cultural stress levels, and shifts in mental health outcomes as individuals navigate the challenges and opportunities associated with immigration. A one-time assessment might miss critical developmental trajectories and fail to capture the dynamic nature of these processes. Moreover, mediation analyses are inherently sensitive to the temporal sequence of events. Cultural stress as a mediator implies a directional relationship where changes in ethnic identity precede changes in cultural stress, which in turn influence mental health outcomes. The single time point may limit the ability to establish a clear temporal sequence necessary for mediation effects to manifest (Cole & Maxwell, 2009). Nonetheless, our study offers a comprehensive overview of the associations among these variables in recently migrated Venezuelan parent–youth dyads, an area that has been largely underexplored.

It is somewhat surprising that ethnic identity was not significantly related either to cultural stress or to mental health. Indeed, prior research with other populations has suggested that ethnic identity may be positively related to experiences of discrimination (Cobb et al., 2017) as well as to mental health (Smith & Silva, 2011). One potential explanation for the lack of similar findings in the present study might involve the sociopolitical situation from which many of the Venezuelan families in our family may have fled. Indeed, Duque et al. (in press) found that Venezuelans in the United States were more likely than Venezuelans in other South American countries to have escaped political persecution. Perhaps ethnic identity may be less strongly linked with cultural stress and mental health in situations where participants left their countries of origin because of traumatic circumstances. Further research is necessary to explore this possibility.

The present results suggest that interventionists and policy makers should focus on promoting national identity among youths and on reducing cultural stress among both youths and parents as ways to improve mental health. Developing activities to promote connection to the United States, for example, might help to increase national identity among youths. Further, given the negative predictive effects of cultural stress on mental health among both youths and parents, it is essential to (a) help these individuals to manage and cope with these stressors and (b) develop social change strategies to reduce discrimination and exclusion toward Venezuelan immigrant families.

## 6. Strengths and Limitations

*Strengths.* Our study includes some notable strengths. The inclusion of a sample comprising 103 Venezuelan parent–youth dyads offers a valuable and specific demographic representation, providing insights into the experiences of recent Venezuelan immigrants in the United States. The emphasis on participants residing in the United States for 36 months or less, with all participants having been in the country for three years or less, provides a timely exploration of the initial phases of immigration and acculturation experiences. Our study also employed a multi-faceted analysis, incorporating actor and partner effects, mediation analysis, and a focus on both ethnic and national identity. This comprehensive approach allows for a nuanced understanding of the psychosocial dynamics within immigrant families.

*Limitations.* The present study is characterized by at least six important limitations. First, the use of a one-time assessment limits the ability to establish causal relationships or capture the dynamic changes in identity, stress, and mental health over time. A longitudinal design would enhance the temporal understanding of these processes. Second, the study’s focus on recently immigrated Venezuelan immigrants may limit the generalizability of findings to broader immigrant populations or those with longer residency in the United States. Third, reliance on self-report measures of ethnic and national identity, cultural stress, and mental health outcomes introduces the potential for bias (e.g., recall bias and social desirability bias) and subjectivity. Additionally, the use of self-report measures of mental health may not capture clinical diagnoses. It is important to include objective measures and clinical diagnostic interviews in future work. Fourth, the study may not capture the full spectrum of cultural and contextual factors (e.g., cultural gaps between parents and adolescents, immigration policies) influencing the experiences of Venezuelan immigrants. Fifth, we were not able to control for key demographic covariates such as immigration status, educational level, socioeconomic bracket, and English proficiency. We therefore do not know the extent to which these variables might have influenced our results. Finally, the focus on Miami as a cultural setting may be both a strength and a limitation. As a strength, Miami is home to many Latin American immigrant groups and provides a supportive context in which to recruit and assess Latin American families. As a limitation, the cultural context of Miami likely does not generalize to other areas of the United States. Miami is one of the few U.S. cities where immigrants and their immediate descendants hold a majority of political and economic power, and where the local population consists of immigrants (and their children and grandchildren) from many different Spanish-speaking backgrounds. It is therefore essential to replicate the present findings in other U.S. contexts.

## 7. Conclusions

In conclusion, the present study stands as a timely and essential contribution to our understanding of the experiences of recent Venezuelan immigrants in the United States. These findings not only deepen our comprehension of the psychosocial experiences faced by Venezuelan immigrant parent–youth dyads, but also contribute to the expanding body of literature on immigrant mental health in general.

Immigrant populations often grapple with multi-faceted challenges during the process of integrating into a new cultural context. Our study emphasizes identity processes and cultural stresses as influential factors shaping the well-being of these families, offering valuable knowledge that can inform targeted interventions, especially for recently immigrated Venezuelan families during their adjustment period. Recognizing both the unique challenges and strengths of the Venezuelan immigrant experience is crucial for designing culturally sensitive support strategies. This contextual awareness is pivotal for the development of interventions that resonate with the lived realities of these families, fostering a more effective and culturally responsive approach to immigrant mental health support. We hope that the present work inspires additional work in this direction.

## Figures and Tables

**Table 1:** Parent–youth mental health model fit through invariance testing.

	RMSEA	CFI	TLI	SRMR	$\chi^{2}$	$\Delta \chi^{2}$	*df*
Configural Model	0.67	1.00	1.00	0.03	5.793		9
Metric Invariance Model	0.90	1.00	1.00	0.03	10.589	5.15	13
Scalar invariance	0.73	1.00	1.00	0.04	25.661	14.46 *	17

* *p* < 0.001.

**Table 2:** APIM-eM mediation model.

Indirect Mediation	β	*p*
Parent Ethnic Identity → Parent Cultural Stress → Youth Mental Health Distress	0.04	0.27
Parent Ethnic Identity → Youth Cultural Stress → Youth Mental Health Distress	0.03	0.73
Youth Ethnic Identity → Youth Cultural Stress → Parent Mental Health Distress	−0.04	0.632
Youth Ethnic Identity → Parent Cultural Stress → Parent Mental Health Distress	−0.04	0.55

## Data Availability

Data Available upon request.
